# Proteome-scale profiling reveals MAFF and MAFG as two novel key transcription factors involved in palmitic acid-induced umbilical vein endothelial cell apoptosis

**DOI:** 10.1186/s12872-021-02246-5

**Published:** 2021-09-17

**Authors:** Mangyuan Wang, Fen Liu, Binbin Fang, Qiang Huo, Yining Yang

**Affiliations:** 1 Clinical Medicine Postdoctoral Research Station, The First Affiliated Hospital of Xinjiang Medical University, 137, Liyushan Road, Xin Shi District, Urumqi, 830054 People’s Republic of China; 2 Department of Cardiac Surgery, The First Affiliated Hospital of Xinjiang Medical University, 137, Liyushan Road, Xin Shi District, Urumqi, 830054 People’s Republic of China; 3 Department of Cardiology, The First Affiliated Hospital of Xinjiang Medical University, 137, Liyushan Road, Xin Shi District, Urumqi, 830054 People’s Republic of China; 4 Xinjiang Key Laboratory of Cardiovascular Disease Research, Urumqi, People’s Republic of China

**Keywords:** Apoptosis, Atherosclerosis, Endothelial cell, HUVEC, Palmitic acid, Transcription factor

## Abstract

**Background:**

Vascular endothelial cell apoptosis is the leading risk factor of atherosclerosis (AS). The purpose of our study was to use a new generation high-throughput transcription factor (TF) detection method to identify novel key TFs in vascular endothelial cell apoptosis induced by palmitic acid (PA).

**Methods:**

Human umbilical vein endothelial cells (HUVECs) were treated with 0, 300, or 500 µM PA. Candidate TFs in the three groups were identified by differential expression, pathway enrichment, Western Blot (WB), and RT-qPCR analyses. Apoptosis was assessed by fluorescence-activated cell sorting (FACS) using FITC-annexin V and propidium iodide staining.

**Results:**

We established a HUVEC apoptosis model to simulate the process of atherosclerosis onset and identified 51 significant TFs. of the 51 TFs, v-maf musculoaponeurotic fibrosarcoma oncogene family protein G (MAFG) and v-maf musculoaponeurotic fibrosarcoma oncogene family protein F (MAFF), were matched to known AS signalling pathways and were validated by WB and RT-qPCR analyses in our study. Overexpression of MAFG or MAFF in HUVECs significantly inhibited PA-induced early apoptosis.

**Conclusions:**

We identified MAFF and MAFG as novel key TFs in vascular endothelial cell apoptosis.

**Supplementary Information:**

The online version contains supplementary material available at 10.1186/s12872-021-02246-5.

## Background

Atherosclerosis (AS) is a common vascular disease characterized by atherosclerotic plaque formation, which is initiated by endothelial cell apoptosis followed by lipid deposition and foam cell formation [1]. AS is the leading cause of cardiac infarction, arterial aneurysm, stroke, and the severe associated complications [2].

Vascular endothelial cell apoptosis is the initial pathogenetic process underlying atherosclerotic plaque formation, and it is the leading risk factor for atherosclerotic plaque formation [1]. To interfere effectively in the early stage of AS and to predict the atherosclerotic plaque formation risk, we focused on identifying the key transcription factors (TFs) in this initial process of AS formation. Human umbilical vein endothelial cells (HUVECs) are cells that recapitulate the characteristics of arterial endothelial cells in vitro. HUVECs with apoptosis induced by 24 h of stimulation with palmitic acid (PA) are a mature cell model of AS [3]. Numerous TFs and signalling pathways, as well as changes in gene expression levels, are likely to be involved in HUVEC apoptosis [4].

TF response element (TFRE) profiling is a new-generation high-throughput TF detection method that allows qualitative and semiquantitative screening of TFs on the proteome scale in specific cells or tissues using nano-liquid chromatography/tandem mass spectrometry (LC–MS) analysis [5]. TFRE profiling provides information that directly reflects changes in TF protein levels as well as the activation state of the TF population.

Although previous studies have reported some TFs that are key players in HUVEC apoptosis [6], high-throughput TF profiling in HUVEC apoptosis models has not yet been reported.

In this study, to identify key TFs involved in HUVEC apoptosis, we conducted TFRE profiling using three experimental groups of HUVECs treated with increasing concentrations of PA to induce apoptosis, and followed with cellular functional validation experiments.

## Methods

### Experimental groups, workflow, and study design of TFRE profiling

HUVECs were grouped as follows: control group; HUVECs treated with 0 µM PA; group 1, HUVECs stimulated with 300 µM PA; and experimental group 2, HUVECs stimulated with 500 µM PA. All three groups were treated with PA for 24 h, and flow cytometry was performed to test the early apoptosis rates among the three groups three times to determine whether the HUVEC apoptosis model to simulate the onset process of atherosclerosis was established. Then, TFRE profiling experiments were performed three times.

### HUVEC cultivation

Primary HUVECs were purchased from COBIOER company (Nanjing, China), and the designated type was primary cells from PromoCell. The cells were checked to ensure that they were free of contamination, and they were used from a low-passage stock. We then cultured the cells with BEGM BulletKit Medium (LONZA, catalogue no. CC-3170). HUVECs were seeded into 18 culture flasks (25 cm^2^ in size), of which three flasks were used for each of the three experimental groups. The cells grew and adhered to the flask walls, and the cell density in each flask was monitored. We added PA to the cell culture when the density was 1 × 10^5^ cells/cm^2^ at 3 days after cell passage. For each group, the six flasks were then combined into three sets of three flasks, which were considered one replicate; the experiment was therefore conducted with three replicates.

SV40T-transformed HUVECs were purchased from ProCell company (catalogue no. CL-0675, Wuhan, China). SV40T-transformed HUVECs were cultured in DMEM-H (Dulbecco’s modified Eagle’s medium + 10% FBS + 1% NEAA) in an incubator at 37 °C with 95% humidity and 5% carbon dioxide. We passaged the cells every 3 days.

### Nuclear protein extraction

A Pierce NE-PER kit (Thermo Scientific, USA) was used to extract the nuclear proteins of the HUVECs. The concentration of nuclear protein was determined by spectrophotometry, and then the volume of nuclear extract was adjusted to guarantee the comparability of protein input among the three groups.

### Enrichment of TFs in nuclear protein extracts using catTFRE

The catTFRE DNA chains were amplified from a plasmid containing catTFRE with biotin-labelled primers (Sigma) via PCR and were bound to Dynabeads M-280 Streptavidin (Invitrogen, USA). The catTFRE-bead complex was then mixed with the nuclear protein extracts and incubated at 4 °C for 2 h to enrich the transcription factors in the nuclear protein extracts. The detailed TFRE procedures are described in our previous study [7].

### Nano-LC–MS/MS analysis for protein identification and label-free quantification

We dissolved dry tryptic peptides in 1% acetonitrile containing 0.1% methanol. Next, we separated one-third of the volume of the peptides on a C18 column, and the peptide samples were analysed on an EASY-nLC 1000 LC system (Thermo Fisher Scientific, Waltham, MA) coupled with Orbitrap Fusion mass spectrometry (Thermo Fisher Scientific, Waltham, MA). MS raw peptide data generated by LC–MS/MS were searched against the UniProt human proteome database (version 2019-03-07, 20, 404 sequences) using MaxQuant (version 1.6.5.0) software (Additional file 8). Finally, we used Proteome Discoverer 1.4 to select TFs from the identified proteins.

### Selection of candidates for key TFs in HUVEC apoptosis

The fraction of total (FOT)-adjusted peak areas were compared among the three groups, and heatmap and volcano plots were drawn based on TF showing variations with both t-test *P* value < 0.05 and fold change > 2. Then, a relational network among these TFs was generated to find the central TFs. Next, we selected differential TFs showing the same variation trends among the three experimental groups. Downstream gene pathway enrichment analysis of the differential TFs with the same variation trends was conducted to select TFs matching known signalling pathways in AS, which were selected as candidate TFs involved in HUVEC apoptosis. Then, we excluded known key TFs involved in AS in other previous studies, and the remaining TFs were selected as candidate novel TFs involved in HUVEC apoptosis after Western Blot (WB) and real-time quantitative PCR (RT-qPCR) verification.

### Western blot (WB) analysis

Total proteins were extracted from HUVECs in the 3 groups. The proteins were separated by SDS-PAGE and transferred to PVDF membranes. Membranes were incubated with the primary antibodies (anti-MAFG, Abcam, ab154318, 1:500; anti-MAFF, Abcam, ab183859, 1:500) overnight at 4 °C after blocking with 5% skim milk for 1 h. Then, membranes were incubated with the secondary antibody (Abcam, ab205718) labelled with horseradish peroxidase. After incubation, we performed washing with TBST (Tris-buffered saline containing Tween 20). The immune complex on the membrane was then visualized by diaminobenzidine. The integrated density was determined by Image Lab™ software version 4.0 (Bio-Rad Laboratories, Inc., Hercules, CA, USA) and normalized to a percentage of the internal control β-actin. Images of original Western Blots were illustrated in Figure S3 (Additional file 3).

### Real time quantitative PCR (RT-qPCR)

Total RNA was extracted from HUVECs with TRIzol reagent. cDNA was synthesized with a reverse transcription kit (QIAGEN, China). RT-qPCR was performed with Power SYBR Green PCR Master Mix (Applied Biosystems, U.S.A.) in a 7900 HT Fast Real-Time PCR system (Applied Biosystems, U.S.A.). The results were calculated with the 2^−ΔΔCt^ method and normalized to a percentage of the internal control of β-actin. The primers used in the present study are listed in Table 1.

**Table 1:** Primers using in RT-qPCR to validate the TFRE results

Molecules	Forward	Reverse
MAFG	TCCAGGGTACTGACCTGCTC	GTTTCCTTTATTGGGGGTCG
MAFF	TGCCCAGGTCCCATTTCTC	GGCCCACGAAGGGAATGT
β-actin	CCTAGAAGCATTTGCGGTGG	GAGCTACGAGCTGCCTGACG

### MAFF or MAFG overexpression (OE) plasmid construction and transfection of SV40T-transformed HUVECs

The plasmid pIRES2-EGFP containing human MAFF cDNA (pMAFF-IRES2-EGFP, Additional file 1: Figure S1) and human MAFG cDNA (pMAFG-IRES2-EGFP, Additional file 2: Figure S2) was constructed by TSINGKE BioTech (Beijing, China). The forward primer for MAFF was CTCGAGATGTCTGTGGATCC, and the reverse primer for MAFF was GAATTCCTAGGAGCAGGA. The forward primer for MAFG was CTCGAGATGACGACCCCCA, and the reverse primer for MAFG was GAATTCCTACGATCGGGC.

After passage for three generations, the cells were transfected for 48 h with MAFF or MAFG OE plasmid or 10 µg of empty plasmid with 10 µl of Lipofectamine 2000 Transfection Reagent (catalogue no.: 11668027, Thermo Fisher Scientific, USA) in each group. We used a fluorescence microscope to observe the GFP signal.

### RT-qPCR and WB validation of MAFF or MAFG OE

RT-qPCR and WB validation of MAFF or MAFG OE was performed in four groups: the control group without plasmid transfection; empty plasmid transfection group; MAFF OE plasmid transfection group; and MAFG OE plasmid transfection group.

The detailed procedures for RT-qPCR and WB analysis are as above in Sects. Western blot (WB) analysis and  Real time quantitative PCR (RT-qPCR). The primers used for validation of MAFF and MAFG OE are listed in Table 2.

**Table 2:** Primers of RT-qPCR to validate the OE of MAFF and MAFG

Molecules	Forward	Reverse
MAFG	AAGGCCTTGAAGGTGAAGCG	CCTTCTGCTTCTCCAGCTCC
MAFF	GAGGAGCTGCAGAAGCAGAA	TGACGATGGTGATGACGCTG
GAPDH	ACAGCAACAGGGTGGTGGAC	TTTGAGGGTGCAGCGAACTT

### Cellular functional validation of MAFF and MAFG and flow cytometric analysis

HUVECs were grouped into 4 experimental groups: the control group (no plasmid and no OE), plasmid group (empty plasmid and no OE), MAFF OE group (MAFF OE plasmid and MAFF OE), and MAFG OE group (MAFG OE plasmid and MAFG OE). The four groups were induced by 500 µM PA for 24 h.

HUVECs were harvested from culture plates using trypsin/EDTA and washed in PBS. Staining was performed for 30 min on ice with FITC-annexin V (30 μg/ml) and propidium iodide (PI, 30 μg/ml, BD Pharmingen, San Diego, CA, USA) in calcium-containing PBS. Fluorescence-activated cell sorting (FACS) analysis was performed immediately (Mindray BriCyte E6 FACS Calibur instrument). A total of 10^5^ cells/sample were counted, and the results were evaluated by using Cell Quest software (Becton Dickinson).

### Statistical analysis

We used the fraction of total (FOT) value to adjust the peak area among groups, and we used Kolmogorov–Smirnov and Shapiro–Wilk tests to test the normality of continuous variables. We used unpaired t-tests to compare continuous variables with a normal distribution. We used the Mann–Whitney U test to compare continuous variables with skewed distributions. A significant difference between two groups was represented by a two-tailed *P* < 0.05. SPSS (version: 20.0) was used to analyse data. GraphPad Prism (version: 6.01) and Adobe Illustrators were used to generate and compose the figures.

## Results

### Establishment of a HUVEC apoptosis model to simulate the onset process of atherosclerosis.

**Fig. 1:**
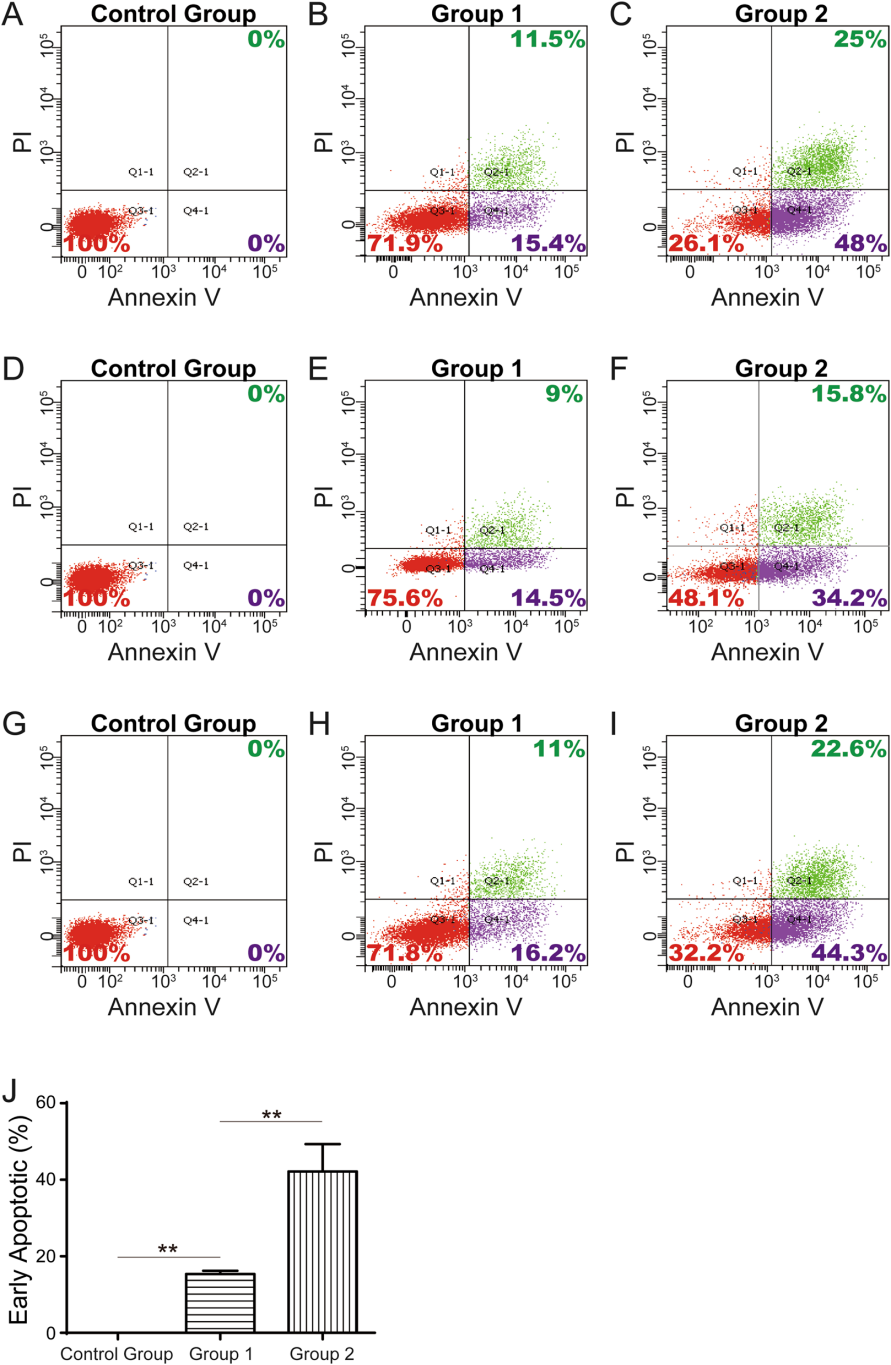
Establishment of the HUVEC apoptosis model to simulate the initiation process of atherosclerosis. Representative flow cytometric analysis showing the apoptosis of HUVECs in the control group (**a**, **d**, **g**), group 1 (**b**, **e**, **h**), and group 2 (**c**, **f**, **i**). **j** Quantitative analysis of the increased early apoptosis percentage in HUVECs treated with various concentrations of PA (n = 3). Control group: 0 μM PA. Group 1: 300 μM PA. Group 2: 500 μM PA. ** indicates 0.001 < *P* < 0.01

We stimulated HUVECs at 300 µM and 500 µM, and flow cytometry was performed to observe the changes in the early apoptosis rate. We found that the early apoptosis rate increased significantly (15.37% ± 0.85% vs 42.17% ± 7.14%, P = 0.022) as the PA concentration increased from 300 µM in group 1 to 500 µM in group 2 (Fig. 1).

### Identification of 51 TFs showing significant differential expression (*P* < 0.05 and fold change > 2)

**Fig. 2:**
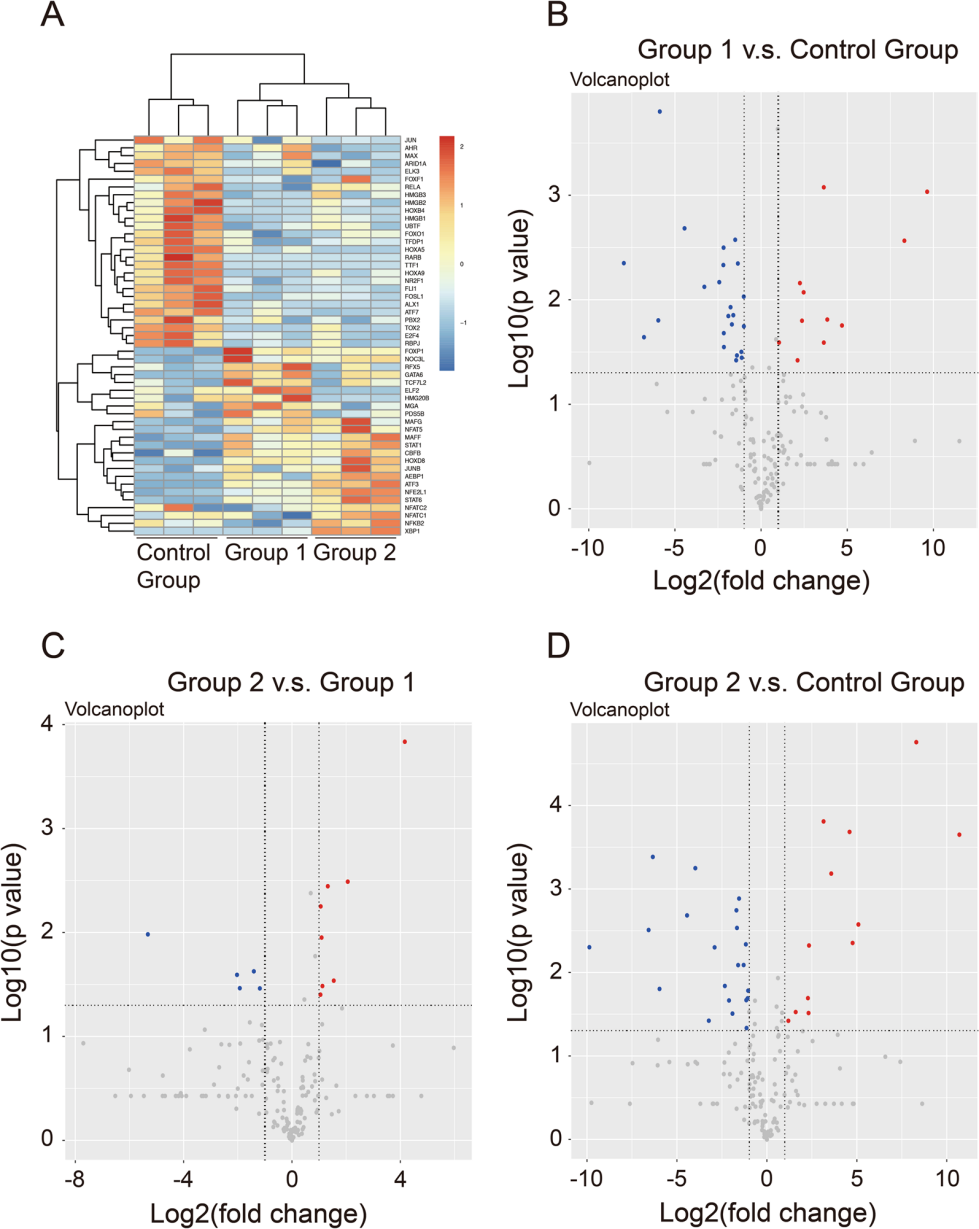
Identification of 51 significant TFs with a t-test *P* value < 0.05 and fold change > 2. **a** The heatmap shows 51 transcription factors (TFs) that showed significant differential expression among the three experimental groups. Blue and red represent low and high expression levels, respectively. **b**–**d** Each dot in the volcano plots shows one TF with both a fold change > 2 and a t-test *P* value < 0.05. The red dots represent upregulated transcription factors and the blue dots represent downregulated transcription factors between two of the three groups

In the three TFRE profiling experiments, we identified 173 TFs in total in the control group, group 1, and group 2. Of these, 36 TFs showed significant differential expression with a t-test *P* value < 0.05 between group 1 and the control group, 34 TFs were significantly differentially expressed between group 2 and the control group, and 13 TFs were significantly differentially expressed between group 2 and group 1. All 51 significant TFs (Additional file 4: Table S1) are shown in Fig. 2a.

As shown in the volcano plots (Fig. 2b–d), 34 TFs (Additional file 5: Table S2) significantly differentially expressed between group 1 and the control group were identified and met the selection criteria of both *P* value < 0.05 and fold change > 2 (Fig. 2b). Of these, 11 TFs were upregulated, shown as red dots in Fig. 2b, and 23 TFs were downregulated, shown as blue dots in Fig. 2b. Thirteen TFs (Additional file 6: Table S3) showing significantly different levels between group 2 and group 1 met the selection criteria (Fig. 2c), of which 8 TFs were upregulated (red dots) and 5 TFs were downregulated (blue dots). Finally, 33 TFs (Additional file 7: Table S4) showing significant differential expression between group 2 and the control group met the selection criteria (Fig. 2d); of these, 12 TFs were upregulated (red dots) and 21 TFs were downregulated (blue dots).

### Protein interaction network analysis and TFs with significant dose-dependent increased or decreased expression between the control group and group 1 and between group 1 and group 2

**Fig. 3:**
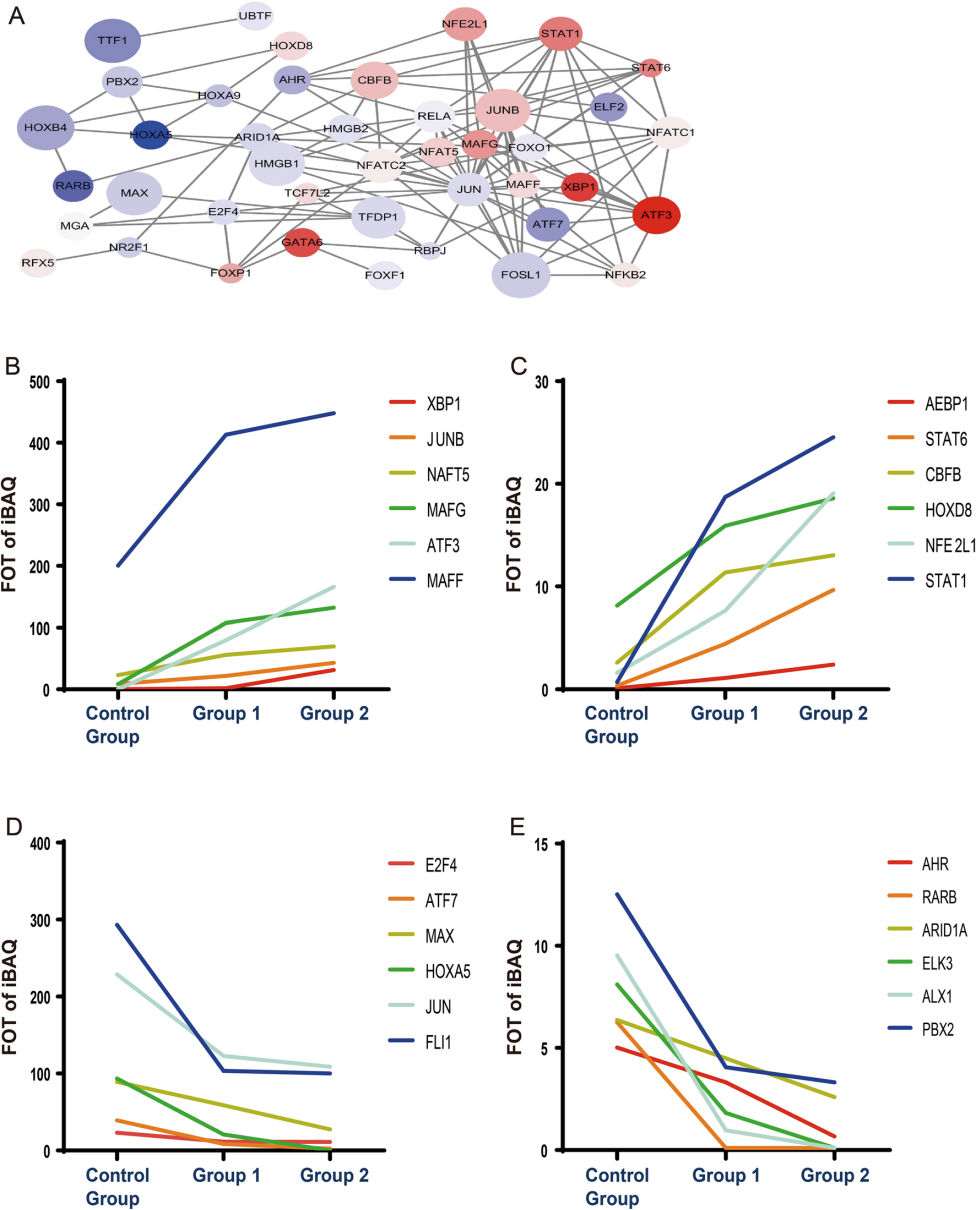
Protein interaction network analysis and differential TFs with increased or decreased expression between the control group and group 2. **a** Relational network among the 51 transcription factors that showed significant differential expression among the three experimental groups. The area of the circle represents the number of interactions of the transcription factor. Red and blue represent upregulation and downregulation, respectively. **b**–**e** Twenty-four differential TFs increased or decreased from the control group to group 2

Protein interaction network analysis (Fig. 3a) revealed that several TFs, including ATF3, JUN, JUNB, MAFG, MAFF, RELA, STAT1, and STAT6, were significantly differentially expressed and interconnected. Among the TFs showing differential expression between the control group and group 1 and between group 1 and group 2 (with dose dependence), 12 of the 51 significant TFs—namely ATF3, XBP1, STAT1, STAT6, AEBP1, MAFG, NFE2L1, CBFB, JUNB, NFAT5, HOXD8, and MAFF—were upregulated in group 1 compared with the control group and further upregulated in group 2 compared with group 1 (Fig. 3b, c), while 12—namely, HOXA5, ALX1, ELK3, RARB, ATF7, AHR, PBX2, MAX, FLI, ARID1A, JUN, and E2F4—were downregulated in group 1 compared with the control group and further downregulated in group 2 compared with group 1 (Fig. 3d, e). Therefore, the expression of these 12 upregulated TFs and 12 downregulated TFs was dose-dependent. Seven TFs overlapped between the significant TFs in the interaction network analysis and the TFs with dose-dependent significantly differential expression in the control group compared with group 1 and in group 1 compared with group 2: ATF3, JUN, JUNB, MAFG, MAFF, STAT1, and STAT6.

### Pathway enrichment of TFs and matching to key signalling pathways of AS; WB and RT-qPCR validation

**Fig. 4:**
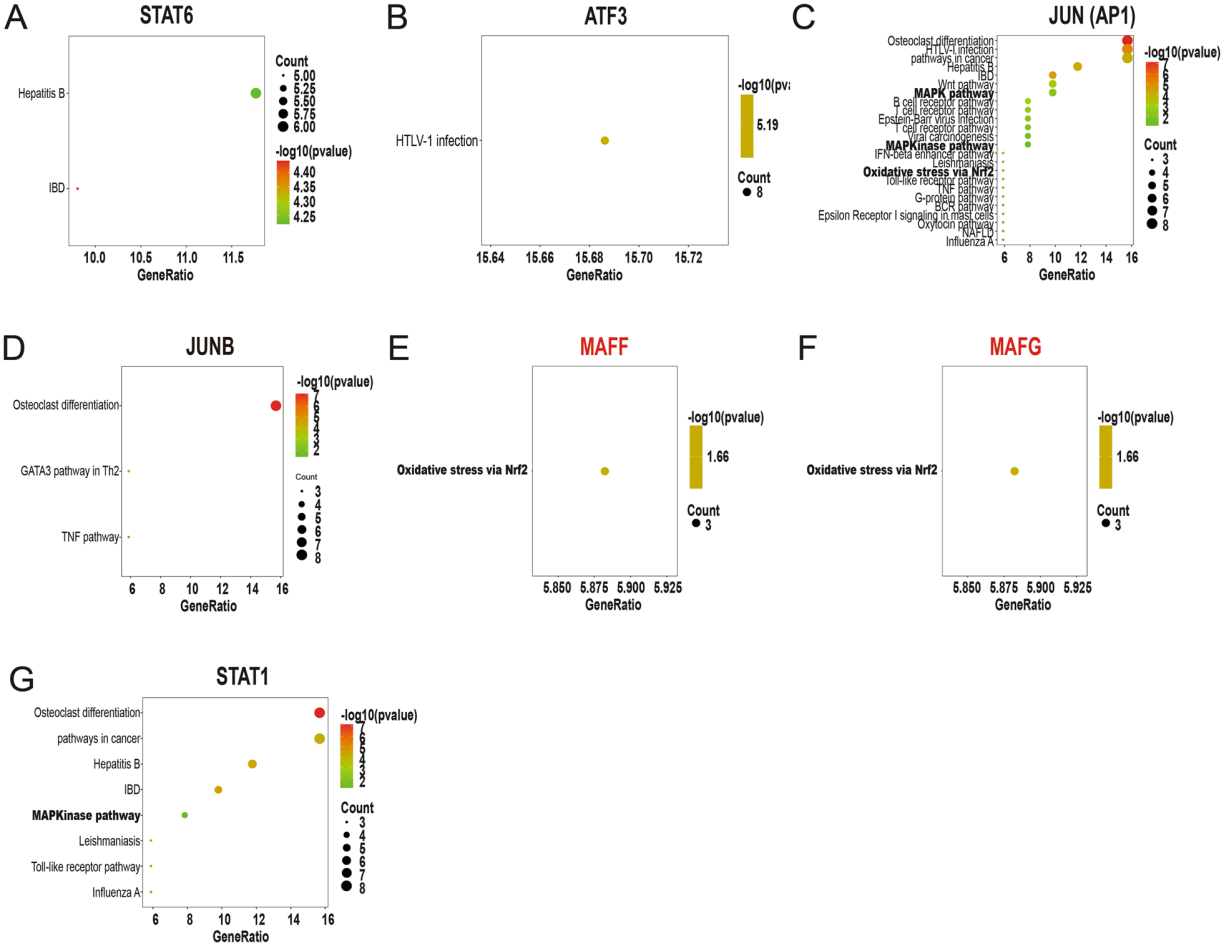
Pathway enrichment of TF downstream genes and matching to key signalling pathways of AS or vascular endothelial cell apoptosis. The transcription factors STAT6 (**a**), ATF3 (**b**), JUN (**c**), JUNB (**d**), MAFF (**e**), MAFG (**f**), and STAT1 (**g**) were enriched in known key signalling pathways related to atherosclerosis. MAFF (**e**) and MAFG (**f**) in red letters are novel candidates of TFs involved in vascular endothelial cell apoptosis and were enriched in known key signalling pathways of atherosclerosis. Signalling pathways with bold letters are known key signalling pathways of atherosclerosis. **e**, **f** The transcription factors indicated with red letters are novel candidate key TFs involved in vascular endothelial cell apoptosis

To identify which of the 7 TFs are correlated with known AS pathways to further narrow the range of the TFRE screening results for novel key TFs involved in AS initiation, we performed downstream pathway enrichment analysis of the 7 overlapping TFs (Fig. 4), and 4 TFs were matched to known key signalling pathways of AS—namely, the NFE2L2 (Nrf2)-ARE, NFKB, and MAPK signalling pathways. The analysis identified four TFs: JUN (AP1) (Fig. 4c), MAFF (Fig. 4e), MAFG (Fig. 4f), and STAT1 (Fig. 4g).

**Fig. 5:**
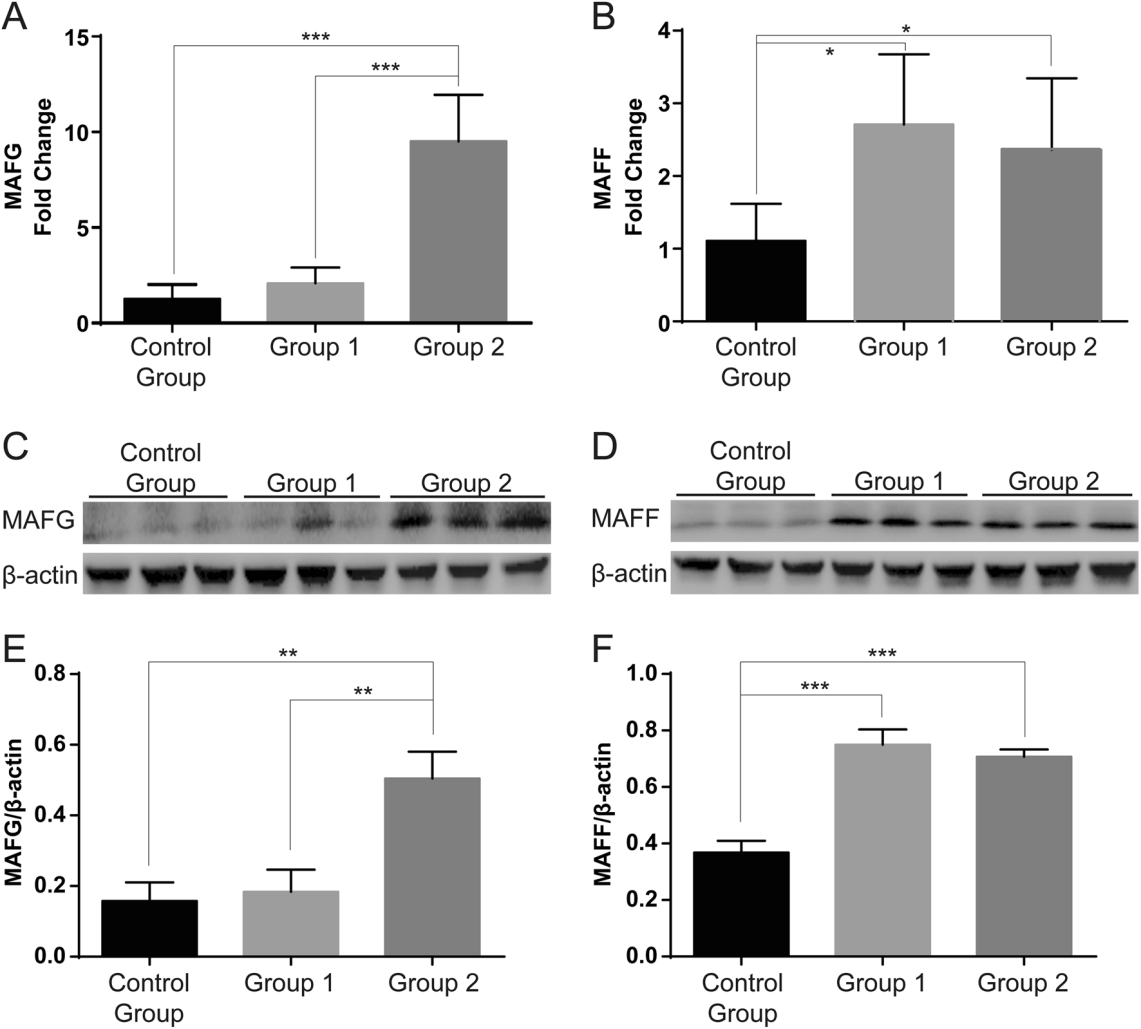
WB and RT-qPCR verification of the two novel candidate key TFs, MAFF and MAFG. **a**, **b** RT-qPCR validation results. **c–f** WB validation results. The change trends in MAFF and MAFG in the RT-qPCR and WB analysis results were consistent with the change trends in the TFRE results, so MAFF and MAFG were validated as two novel candidate key TFs in AS. * indicates 0.01 < *P* < 0.05, ** indicates 0.001 < *P* < 0.01, and *** indicates *P* < 0.001

We identified four candidate TFs—JUN (AP1), MAFG, MAFF, and STAT1—in the previous “Protein interaction network analysis and TFs with significant dose-dependent increased or decreased expression between the control group and group 1 and between group 1 and group 2” section, and two of the four TFs, JUN and STAT1, were then found to be two known key TFs in AS in other former studies by searching articles [8, 9]. Therefore, the remaining two novel candidate key TFs, MAFF and MAFG, were verified by performing WB analysis and RT-qPCR. The results of RT-qPCR (Fig. 5a, b) and WB analysis (Fig. 5c–f) showed that the change trends in these two TFs were consistent with the change trends in the TFRE results; thus, MAFF and MAFG were verified as two novel candidate key TFs in AS.

### Alleviation of apoptosis induced by PA in SV40T-transformed HUVECs with MAFF and MAFG overexpression (OE)

**Fig. 6:**
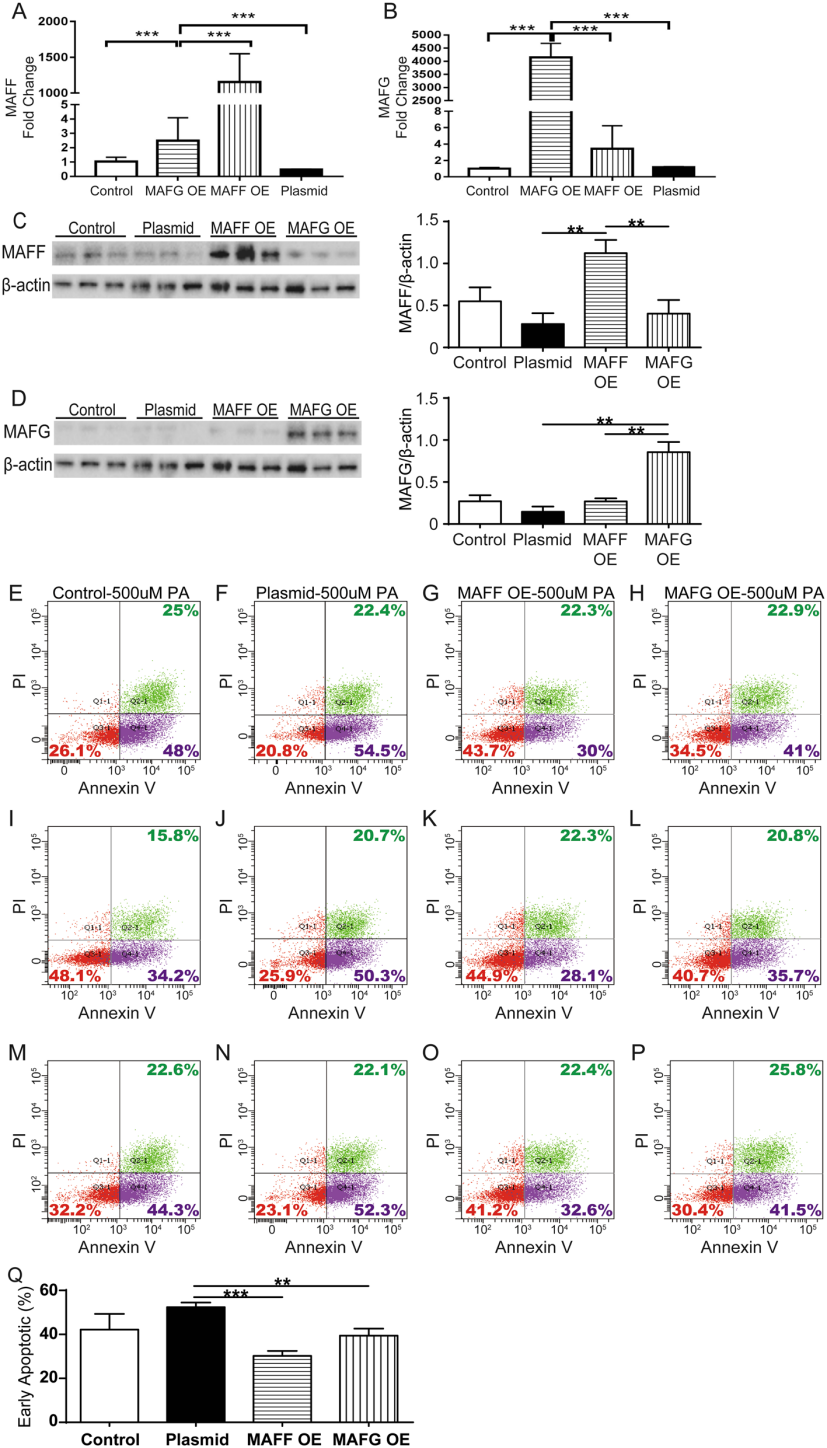
Alleviation of early apoptosis induced by PA in SV40T-transformed HUVECs overexpressing MAFF and MAFG. **a** RT-qPCR validation of MAFF OE. **b** RT-qPCR validation of MAFG OE. **c** WB validation of MAFF OE. **d** WB validation of MAFG OE. **e–h** The first apoptosis assay by flow cytometry. **i**–**l** The second apoptosis assay by flow cytometry. **m**–**p** the third time of apoptosis assay by flow cytometry. **e**, **i**, **m** Control-500 μM PA group subjected to three independent apoptosis assays by flow cytometry. **f**, **j**, **n** Plasmid-500 μM PA group subjected to three independent apoptosis assays by flow cytometry. **g**, **k**, **o** Results for the MAFF OE-500 μM PA group in the three independent apoptosis assays by flow cytometry. **h**, **l**, **p** Results for the MAFG OE-500 μM PA group in the three independent apoptosis assays by flow cytometry. **q** Histogram summary results in the four groups. The dots located in Q4 represent early apoptotic cells. OE represents overexpression. ** indicates 0.001 < *P* < 0.01 and *** indicates *P* < 0.001. Control-500 μM PA: without transfection and with 500 μM PA stimulation. Plasmid-500 μM PA: with empty plasmid transfection and with 500 μM PA stimulation. MAFF OE-500 μM PA: MAFF OE plasmid transfection and 500 μM PA stimulation. MAFG OE-500 μM PA: MAFG OE plasmid transfection and 500 μM PA stimulation

To validate the roles of the novel candidate TFs, we performed biological cell functionality validation experiments to reveal whether MAFF and MAFG can inhibit the PA-induced apoptosis of SV40T-transformed HUVECs. HUVECs were grouped into 4 experimental groups: the control group (no plasmid), empty plasmid group, MAFF OE group, and MAFG OE group. The RT-qPCR and WB analysis results showed that MAFF and MAFG were indeed significantly overexpressed in the MAFF OE group and MAFG OE group, respectively. The four groups were induced by 500 µM PA for 24 h. RT-qPCR and WB analysis results showed that MAFF and MAFG were overexpressed in the MAFF OE group and MAFG OE group, respectively (Fig. 6a–d). The flow cytometry results (Fig. 6e–p) showed that early apoptosis was significantly inhibited in HUVECs with MAFF OE (30.23% ± 2.26% vs 52.37% ± 2.10%, *P* < 0.001) or MAFG OE (39.4% ± 3.21% vs 52.37% ± 2.10%, *P* = 0.019) compared with HUVECs transfected with empty plasmid (Fig. 6q).

## Discussion

### Major findings

First, we established a HUVEC apoptosis model to simulate the onset process of atherosclerosis. Then, we identified 51 TFs that showed significant differential expression with a t-test *P* value < 0.05 and fold change > 2. Then, seven overlapping TFs between the significantly differentially regulated and interconnected TFs and the differentially expressed TFs between the control group and group 2 (with dose dependence) were identified, four of which were found to have downstream genes matching known signalling pathways of AS. The involvement of two of the four TFs—MAFF and MAFG—was still unknown in AS, and the WB and RT-qPCR verification results demonstrated that these two TFs were two novel candidate key TFs in AS. The biological cellular functionality validation results revealed that MAFF and MAFG could significantly inhibit PA-induced early apoptosis of SV40T-transformed HUVECs.

### Significance of this study

Numerous TFs are involved in vascular endothelial cell apoptosis, which is the key process underlying AS onset. However, proteome-scale transcriptional profiling to identify TFs associated with this key event in AS pathogenesis has never been performed. Our results revealed two novel key TFs. These 2 novel key TFs involved in vascular endothelial cell apoptosis are probably protective TFs for AS by inhibiting apoptosis of arterial endothelial cells, and they may be useful for early-stage medical intervention in AS.

### Comparison with other relevant studies and deductions from major results

Several studies have reported key TFs in AS, which were validated by observing changes in the atherosclerotic plaque area and thickness after manipulation of TF gene expression in an ApoE–mouse AS model [6, 10–27]. In this study, we also identified some of these key TFs, such as ATF3 and JUN (AP1). In addition, ALX1, ELK3, RARB, ATF7, MAFG, NFE2L1, CBFB, PBX2, MAX, FLI1, ARID1A, and MAFF were newly identified TFs reported in our present study as key TFs in AS.

According to the Genome Informatics database, all 24 known and unknown TFs identified here are involved in AS-related biological processes, such as cell proliferation and apoptosis. Our results showed that 4 candidate TFs were upregulated from the control group to group 2 in a dose-dependent manner. Considering that the concentration of PA was increased from 0 to 500 µM between the control group and group 2, the upregulation of the 4 TFs in group 2 compared to the control group suggests a relevant association of these TFs with vascular endothelial cell apoptosis, making them attractive targets for early therapeutic intervention. The key signalling pathways of AS on which current research has focused are the NFE2L2-ARE, MAPK, and NFKB pathways [28, 29]. In addition, pathway enrichment for downstream genes of JUN (AP1), MAFG, MAFF, and STAT1 showed an association with key AS signalling pathways. However, JUN (AP1) and STAT1 had already been biologically validated as key TFs in AS in a previous study [8, 9]. Therefore, we deduced from our results that MAFF and MAFG might play a key role in HUVEC apoptosis via the NFE2L2-ARE signalling pathway, because transcription factors MAFF and MAFG were done pathway enrichment to NFE2L2, which is the key molecule of the NFE2L2-ARE signalling pathway. Our results demonstrated that MAFF and MAFG are two novel key TFs in vascular endothelial cell apoptosis.

The functions of v-maf musculoaponeurotic fibrosarcoma oncogene family protein G (MAFG) and v-maf musculoaponeurotic fibrosarcoma oncogene family protein F (MAFF) are now known as proliferation and differentiation coactivators acting in cooperation with NFE2L2 in the mouse hepatoma cell line Hepa1c1c7 (Hepa1) via the Keap1-NFE2L2 pathway [30], and the protein MAFG acts in cooperation with NFE2L2 (Nrf2) to transcriptionally govern either repressive or activating gene functions in antioxidant response element (ARE)-dependent antioxidant pathways in skeletal muscle cells [31]. The protein MAFG is also involved in bile acid homeostasis [32], liver cancer[33], osteosarcoma[34], and central nervous system inflammation[35]. Von Scheidt M et al.[36] demonstrated that MAFF is a novel central regulator of an atherosclerosis-relevant liver network and that MAFF can trigger context-specific expression of LDLR and other genes known to affect coronary artery disease risk. Our research team has published several articles in this field demonstrating that the transcription factors STAT3 and NF-kappaB are involved in atherosclerosis pathogenesis [37–40].

However, NFE2L1 was significantly increased between the control group and experimental groups compared with NFE2L2 in our study, accompanied by a significant increase in MAFF and MAFG. Therefore, we speculate that MAFF, MAFG, and NFE2L1 can transcriptionally activate ARE-associated genes collaboratively in arterial endothelial cells, and the expression of these ARE-associated genes leads to antioxidation enhancement of arterial endothelial cells. Enhancement of antioxidation and reduction in cell injury in the arterial endothelium play crucial roles in atherogenesis suppression. Here we indicated that both MAFF and MAFG could protect HUVECs from apoptosis, indicating that these two TFs might be effective targets for AS treatment.

### Limitations

First, TFRE profiling is a semiquantitative method with limited accuracy. Second, the novel key TFs need to be validated experimentally in the ApoE-/- mouse AS model, and this could be achieved in a future study.

## Conclusion

Here, we identified two novel key TFs, MAFF and MAFG, involved in PA + glucose-induced vascular endothelial cell apoptosis, which is a key event underlying the onset of AS. These results need to be further biologically validated in the ApoE-/- mouse AS model in the future (Additional file 3).

## Supplementary Information

**Additional file 1**: **Figure S1** Schematic diagram of plasmid construction. Plasmid pIRES2-EGFP containing human MAFF cDNA (pMAFF-IRES2-EGFP).

**Additional file 2**: **Figure S2** Plasmid construction schematic diagram. Plasmid pIRES2-EGFP containing human MAFG cDNA (pMAFG-IRES2-EGFP).

**Additional file 3**: **Figure S3** Images of original Western Blots. (A) The original Western Blots image of MAFF blots in Figure 5D. (B) The original Western Blots image of β-actin blots in Figure 5D. (C) The original Western Blots image of MAFG blots in Figure 5C. (D) The original Western Blots image of β-actin blots in Figure 5C. (E) The original Western Blots image of MAFF blots in Figure 6C. (F) The original Western Blots image of MAFG blots in Figure 6D. (G) The original Western Blots image of β-actin blots in Figure 6C and 6D.

**Additional file 4**: **Table S1** TFRE results. Significantly different transcription factors among the control group, group 1, and group 2.

**Additional file 5**: **Table S2** TFRE results. Significantly different transcription factors between group 1 and the control group met the selection criteria of both a P value <0.05 and a fold change >2

**Additional file 6**: **Table S3** TFRE results. Significantly different transcription factors between group 2 and group 1 met the selection criteria of both P value <0.05 and fold change >2

**Additional file 7**: **Table S4** TFRE results. Significantly different transcription factors between group 2 and the control group met the selection criteria of both a P value <0.05 and a fold change >2

**Additional file 8**: Raw TFRE data. Raw proteome-scale profiling data.

## Data Availability

The first author can be contacted if the raw data are needed. The email address of the first author is wangmangyuan2013@163.com.

## Abbreviations

AS: Atherosclerosis
HUVEC: Human umbilical vein endothelial cell
PA: Palmitic acid
TFs: Transcription factors
TFRE: TF response element
LC-MS: Nano-liquid chromatography/tandem mass spectrometry
MAFG: V-maf musculoaponeurotic fibrosarcoma oncogene family protein G
MAFF: V-maf musculoaponeurotic fibrosarcoma oncogene family protein F
WB: Western blot
RT-qPCR: Real-time quantitative PCR
OE: Overexpression
