# Supplementary figures and images for: Proteome-scale profiling reveals MAFF and MAFG as two novel key transcription factors involved in palmitic acid-induced umbilical vein endothelial cell apoptosis
Source: BMC Cardiovasc Disord. 2021 Sep 17;21:448. doi: 10.1186/s12872-021-02246-5 (PMC8447594; doi:10.1186/s12872-021-02246-5)

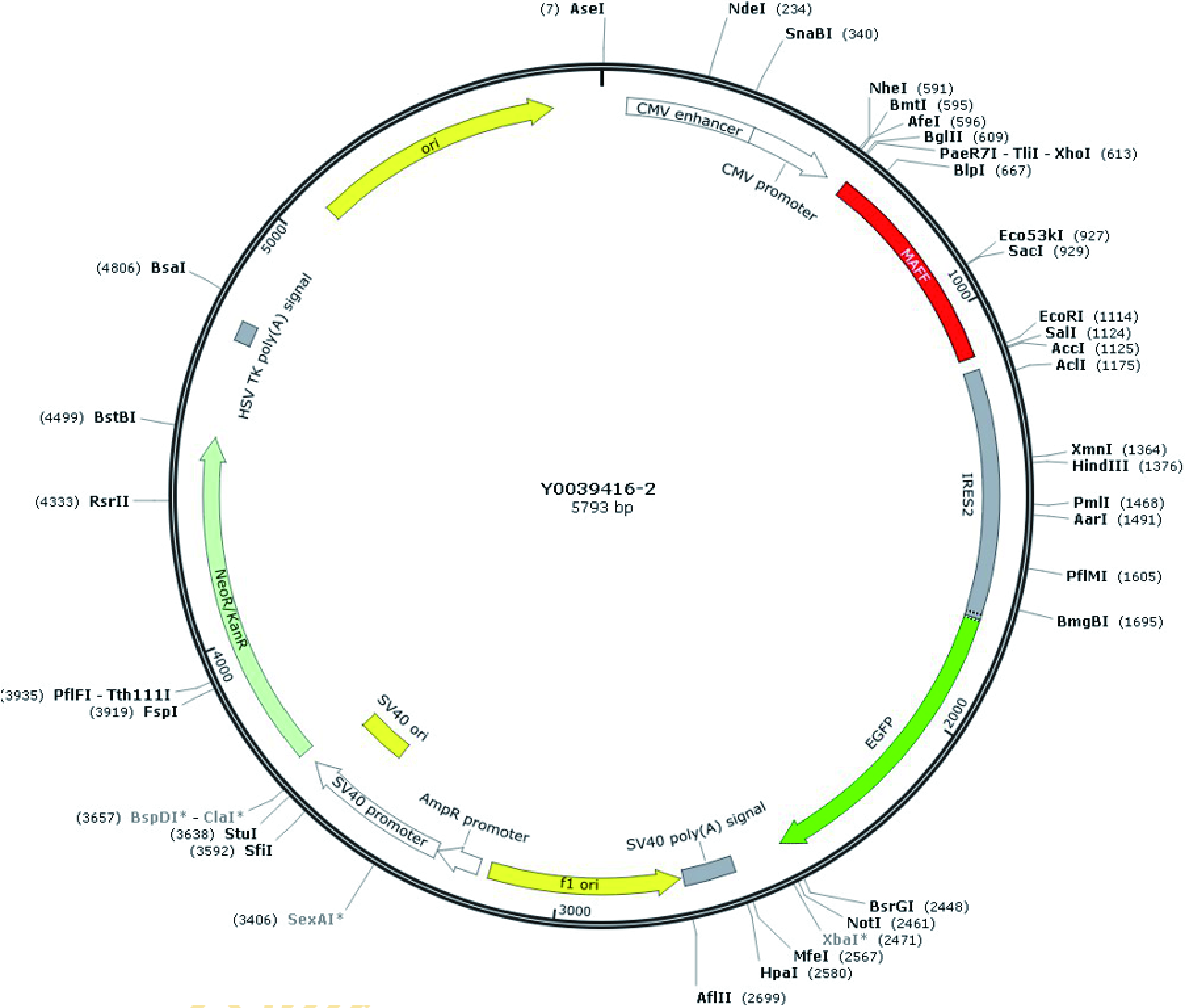

Supplement: Supplementary file 1 — Additional file 1: Figure S1 Schematic diagram of plasmid construction. Plasmid pIRES2-EGFP containing human MAFF cDNA (pMAFF-IRES2-EGFP). [file 12872_2021_2246_MOESM1_ESM.tif]

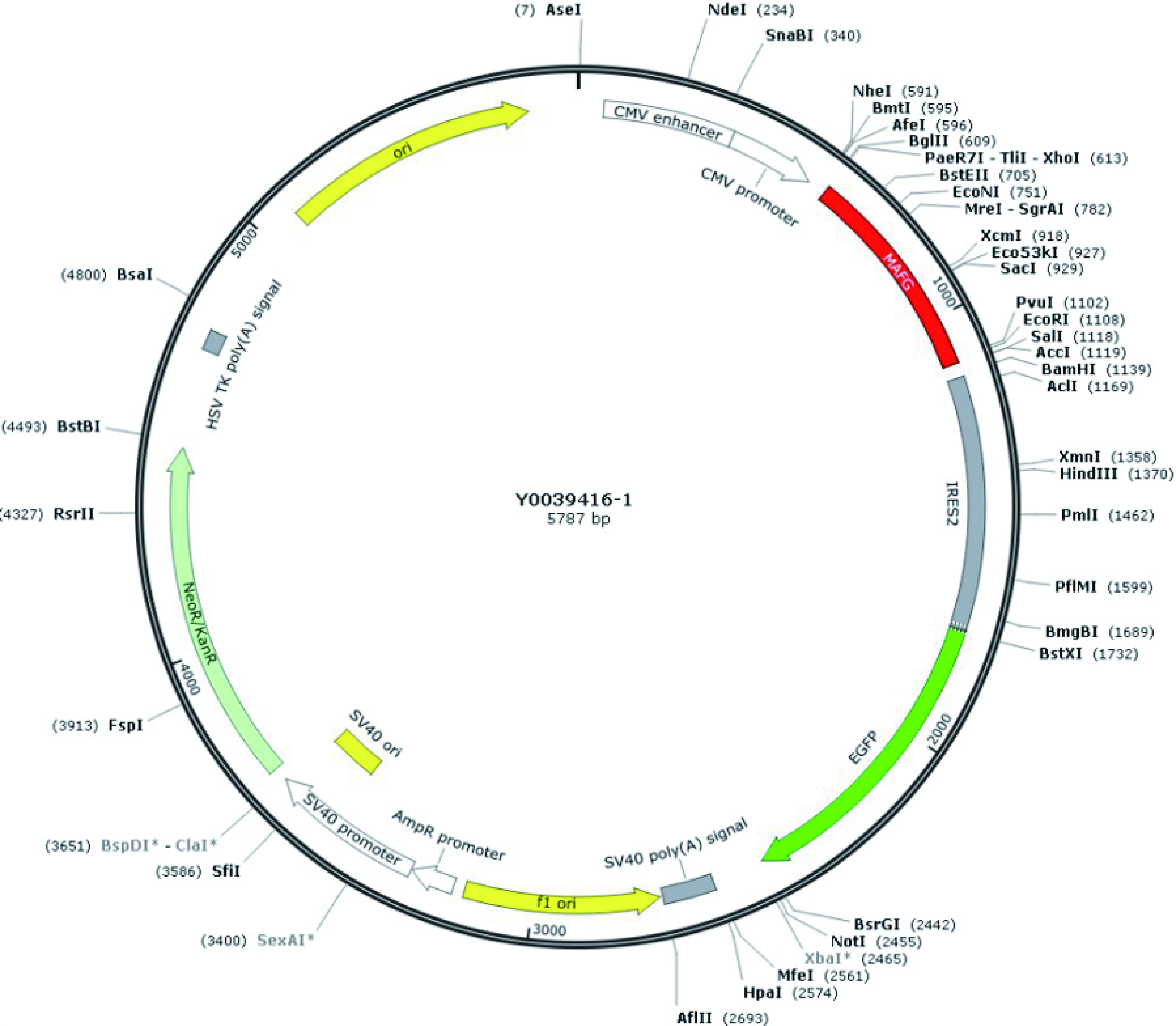

Supplement: Supplementary file 2 — Additional file 2: Figure S2 Plasmid construction schematic diagram. Plasmid pIRES2-EGFP containing human MAFG cDNA (pMAFG-IRES2-EGFP). [file 12872_2021_2246_MOESM2_ESM.tif]

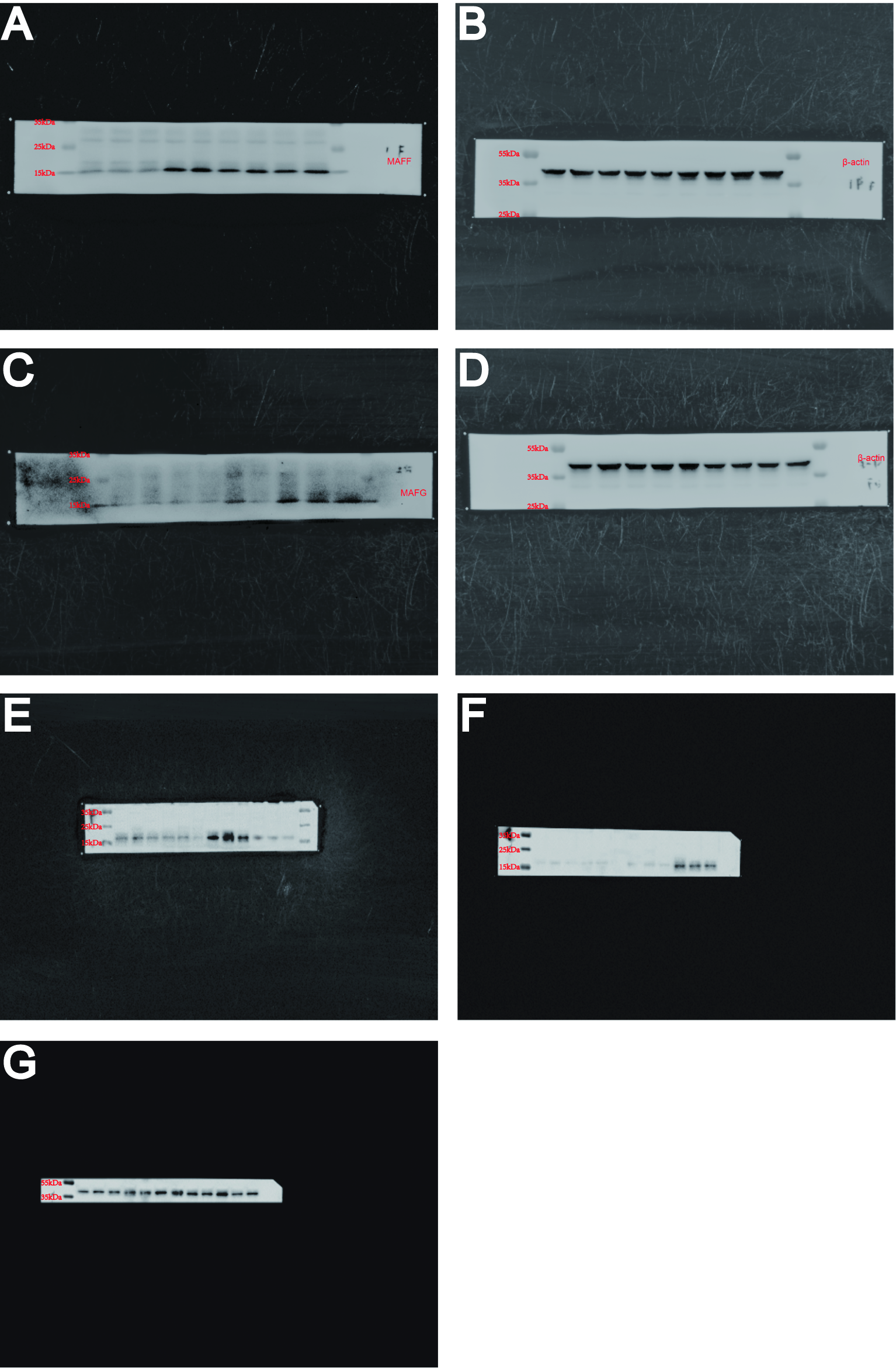

Supplement: Supplementary file 3 — Additional file 3: Figure S3 Images of original Western Blots. (A) The original Western Blots image of MAFF blots in Figure 5D. (B) The original Western Blots image of β-actin blots in Figure 5D. (C) The original Western Blots image of MAFG blots in Figure 5C. (D) The original Western Blots image of β-actin blots in Figure 5C. (E) The original Western Blots image of MAFF blots in Figure 6C. (F) The original Western Blots image of MAFG blots in Figure 6D. (G) The original Western Blots image of β-actin blots in Figure 6C and 6D. [file 12872_2021_2246_MOESM3_ESM.tif]
